# Hypoxic and Hypercapnic Responses in Transgenic Murine Model of Alzheimer’s Disease Overexpressing Human AβPP: The Effects of Pretreatment with Memantine and Rivastigmine

**DOI:** 10.3390/ijms23116004

**Published:** 2022-05-26

**Authors:** Kryspin Andrzejewski, Monika Jampolska, Ilona Mojzych, Silvia V. Conde, Katarzyna Kaczyńska

**Affiliations:** 1 Department of Respiration Physiology, Mossakowski Medical Research Institute, Polish Academy of Sciences, Pawińskiego 5 St., 02-106 Warsaw, Poland; kandrzejewski@imdik.pan.pl (K.A.); mjampolska@imdik.pan.pl (M.J.); ilona.mojzych@student.uw.edu.pl (I.M.); 2 Department of Chemistry, University of Warsaw, Pasteura 1, 02-093 Warsaw, Poland; 3 Faculdade de Ciencias Médicas, NOVA Medical School, Universidade NOVA de Lisboa, 1169-056 Lisbon, Portugal; silvia.conde@nms.unl.pt

**Keywords:** Alzheimer’s disease, transgenic mouse model, breathing, hypoxia, hypercapnia

## Abstract

Despite the severe respiratory problems reducing the quality of life for Alzheimer’s disease (AD) patients, their causes are poorly understood. We aimed to investigate hypoxic and hypercapnic respiratory responses in a transgenic mouse model of AD (AβPP V717I) overexpressing AβPP and mimicking early-onset AD. The cholinesterase inhibitor rivastigmine and the NMDA receptor antagonist memantine were used to investigate the effects of drugs, used to treat AD cognitive dysfunction, on breathing in hypoxia and hypercapnia. We found a significant increase in the respiratory response to hypercapnia and no difference in the hypoxic response in APP+ mice, compared with the control group (APP−). Memantine had no effect on respiration in either group, including responses to hypoxia and hypercapnia. Rivastigmine depressed resting ventilation and response to hypercapnia irrespective of the mice genotype. Reduction in hypoxia-augmented ventilation by rivastigmine was observed only in APP+ mice, which exhibited lower acetylcholinesterase activity in the hippocampus. Treatment with rivastigmine reduced the enzyme activity in both groups equally in the hippocampus and brainstem. The increased ventilatory response to hypercapnia in transgenic mice may indicate alterations in chemoreceptive respiratory nuclei, resulting in increased CO_2_ sensitivity. Rivastigmine is a potent reductant of normoxic and hypercapnic respiration in APP+ and APP− mice.

## 1. Introduction

Alzheimer’s disease (AD), an age-related neurodegenerative disorder, is the most common cause of dementia, affecting more than 45 million people worldwide. Its main features are cognitive and neuropsychiatric symptoms, leading to progressive impairment and disability [1,2,3]. Neurodegeneration, decreased synaptic density, and depletion of brain neurotransmitters are considered to be consequences of pathological processes determined by progressive deposition of amyloid-β (Aβ) plaques and intracellular neurofibrillary tangles of hyperphosphorylated tau protein [4,5,6,7]. In addition to cognitive impairment, respiratory distress is common in AD patients. Among respiratory disorders, sleep apnea takes the lead, but respiratory dysrhythmias, dyspnea, respiratory muscle strength decline, bronchiolitis, and pneumonia are also observed [8,9,10,11], and while they can indeed reduce the quality of life, they are often undiagnosed and untreated, and their causes are not well-understood. Respiratory responses to hypoxia or hypercapnia have not yet been studied in patients with AD, although maladaptation of respiratory responses to these stimuli occurring during apnea may lead to impaired respiratory gas homeostasis and compatible cellular homeostasis.

Recently, attempts have been made to investigate respiratory changes in an animal model of the disease induced by injection of streptozotocin (STZ) into the lateral ventricles of the rat brain, mimicking sporadic AD. Unfortunately, the results are inconclusive and vary between studies, despite fairly similar experimental conditions. Ebel et al. [12] and Brown et al. [13] showed blunted ventilatory response to hypoxia, while Vincente et al. [14] displayed increased sensitivity to hypercapnia.

In our search for a suitable model to study respiratory impairment in Alzheimer’s disease, we examined hypoxic and hypercapnic respiratory responses, for the first time, in transgenic mice using the AβPP V717I model, a “London mutation” characterized by mutation of the APP gene and significant overexpression of AβPP. The same mutation is present in familial AD with early onset, and the model is well-known to mimic the behavioral, biochemical, or electrophysiological changes that characterize AD [15,16]. In further steps, we examined whether drugs used as standard treatment for cognitive impairment in AD have an effect on breathing, which has not been studied before. In our study, we applied a cholinesterase inhibitor, rivastigmine, or an N-methyl-D-aspartate (NMDA) receptor antagonist, memantine, before hypoxia or hypercapnia exposure. Additionally, to test whether APP+ transgenic mice exhibit abnormalities in the cholinergic system, we assessed brain acetylcholinesterase (AChE) activity in the cerebral cortex, hippocampus, and brainstem, a key region containing nuclei that regulate respiration. The effect of rivastigmine administration on AChE activity was also studied.

## 2. Results

### 2.1. Respiratory Responses to Hypoxia and Hypercapnia

**Figure 1:**
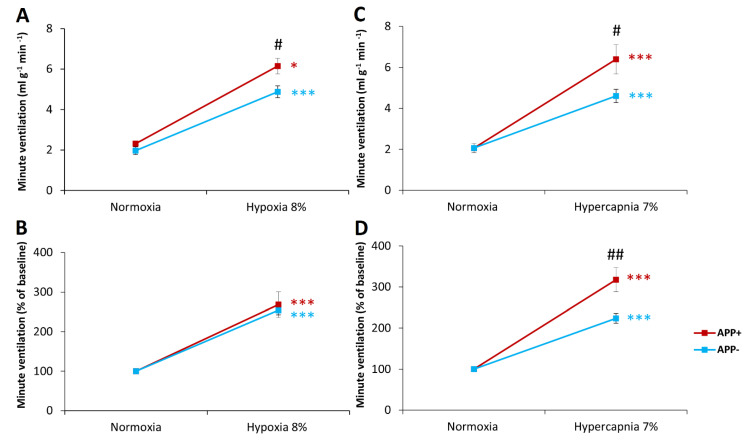
Minute ventilation (V_E_) during air breathing (normoxia) and ventilatory response to hypoxia and hypercapnia in APP+ (red line) and APP− mice (blue line) (**A**,**B**). Minute ventilation reactivity to hypoxia and hypercapnia expressed as a percentage of baseline (normoxia) values (**B**,**D**). Note the lack of difference in hypoxia reactivity in the two groups of mice (**B**) and significant increase in respiratory response and reactivity to hypercapnia in APP+ mice (**C**,**D**). The data are presented as mean ± SEM; * *p* < 0.05. *** *p* < 0.001 vs. normoxia value. # *p* < 0.05, ## *p* < 0.01 vs. APP− group (n = 6 for APP+ and APP−).

Baseline respiratory parameters during air breathing achieved similar values in both APP+ and APP− mice (Table 1, Figure 1A,C). There was a trend toward increased tidal volume (V_T_) in APP+ mice but did not reach statistical significance. Three-minute exposure to hypoxia (8% O_2_ in N_2_) evoked significant respiratory stimulation. Although APP+ mice exhibited increased minute ventilation (V_E_), compared with APP− mice (Figure 1A), mostly as a result of significantly increased frequency of breathing (F), during hypoxic stimulation (Table 1), hypoxia reactivity calculated as a percent of baseline respiration did not differ between the study groups (Figure 1B, Table 1).

The hypercapnic stimulus (7% CO_2_ in oxygen) enhanced ventilation by elevating all respiratory parameters—V_T_, F, and V_E_ (Table 1, Figure 1C). However, the increase in minute ventilation after hypercapnic exposure was greater in APP+ mice than in APP− mice (Figure 1C). This increase was also observed when the data were converted as a percentage of the increase with respect to normoxic respiration, i.e., in actual hypercapnia reactivity. This increase in V_E_ during hypercapnia was mainly due to a greater increase in respiratory rate response (Table 1).

### 2.2. Respiratory Responses to Hypoxia and Hypercapnia after Memantine Administration

**Figure 2:**
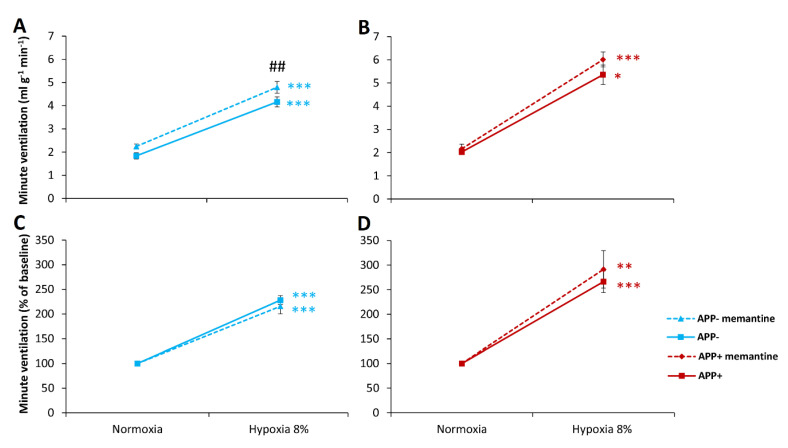
Minute ventilation (V_E_) during air breathing (normoxia) and ventilatory response to hypoxia in APP+ (red line) and APP− mice (blue line) before (solid line) and after memantine injection (dashed line) (**A**,**B**). Minute ventilation expressed as a percentage of baseline (normoxia) values (**C**,**D**). Note the lack of difference in hypoxia reactivity in the two groups of mice. The data are presented as mean ± SEM; * *p* < 0.05, ** *p* < 0.01, *** *p* < 0.001 vs. normoxia value, ## *p* < 0.001 vs. pre-memantine state (n = 6 for APP+ and APP−).

Memantine, an NMDA receptor antagonist, had no significant effect on normoxic minute ventilation (Figure 2A,B) and other ventilation parameters (Table 2) in APP− and APP+ animals. In both genotypic conditions, memantine did not affect the ventilatory response to hypoxia (Figure 2, Table 2).

**Figure 3:**
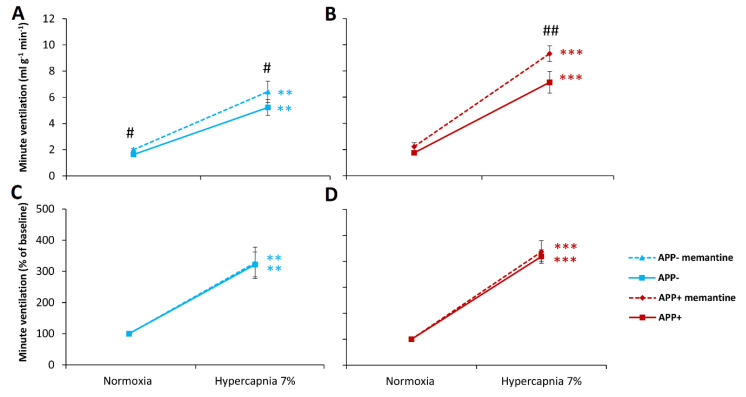
Minute ventilation (V_E_) during air breathing (normoxia) and ventilatory response to hypercapnia in APP+ (red line) and APP− mice (blue line) before (solid line) and after memantine injection (dashed line) (**A**,**B**). Minute ventilation expressed as a percentage of baseline (normoxia) values (**C**,**D**). Note the lack of difference in hypercapnia reactivity in the two groups of mice. The data are presented as mean ± SEM, ** *p* < 0.01, *** *p* < 0.001 vs. normoxia value, # *p* < 0.05. ## *p* < 0.01 vs. pre-memantine state (n = 6 for APP+ and APP−).

This time memantine showed a mild stimulatory effect on normoxic respiration in APP− group (Table 3, Figure 3A). Memantine affected neither hypercapnic stimulation of tidal volume nor the frequency of breathing (Table 3). A significant difference between APP+ and APP− mice in the frequency response confirmed results described in Section 2.1—namely, that APP+ mice presented a higher response to CO_2_ stimulus. The hypercapnic minute ventilation response after memantine administration was manifested by a comparable increase in both groups of mice (Figure 3A,B). However, respiratory reactivity to hypercapnia after memantine administration remained unchanged in both groups of mice (Figure 3C,D, Table 3).

### 2.3. Respiratory Responses to Hypoxia and Hypercapnia after Rivastigmine Administration

**Figure 4:**
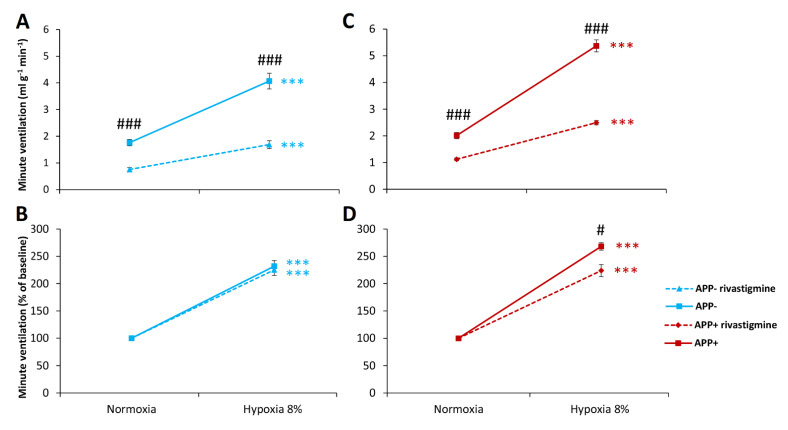
Minute ventilation (V_E_) during air breathing (normoxia) and ventilatory response to hypoxia in APP+ (red line) and APP− mice (blue line) before (solid line) and after rivastigmine injection (dashed line) (**A**,**B**). Minute ventilation expressed as a percentage of baseline (normoxia) values (**C**,**D**). Note the significant decline of reactivity to hypoxia evoked by rivastigmine only in APP+ mice. The data are presented as mean ± SEM; *** *p* < 0.001 vs. normoxia value. # *p* < 0.05, ### *p* < 0.001 vs. pre-rivastigmine state (n = 6 for APP+ and APP−).

Rivastigmine treatment had an effect on resting minute ventilation, which decreased by 44% in APP+ mice and 57% in APP− mice (Figure 4A,C). These changes were mainly due to a decrease in V_T_ (Table 4).

The ventilatory response to hypoxia after rivastigmine was suppressed for V_E_ (54% APP+; 58% APP−), V_T,_ and F (Table 4), but reactivity to the hypoxic stimulus was significantly reduced only for V_E_ in APP+ mice and reached levels present in APP− mice before and after rivastigmine administration (Figure 4B,D).

**Figure 5:**
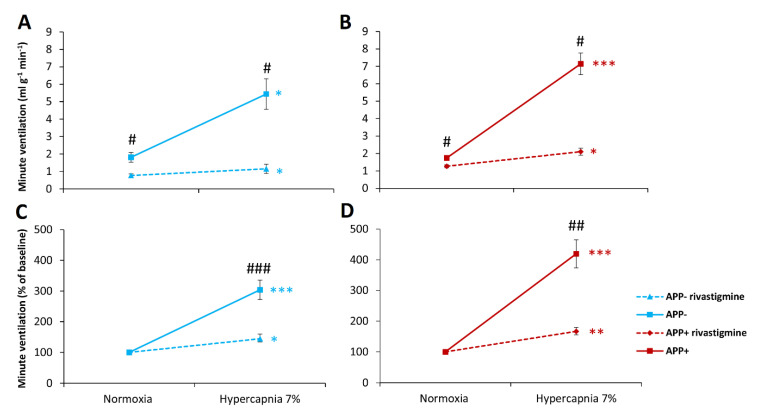
Minute ventilation (V_E_) during air breathing (normoxia) and ventilatory response to hypercapnia in APP+ (red line) and APP− mice (blue line) before (solid line) and after rivastigmine injection (dashed line) (**A**,**B**). Minute ventilation expressed as a percentage of baseline (normoxia) values (**C**,**D**). Note the significantly reduced hypercapnia reactivity in both groups of mice. The data are presented as mean ± SEM; * *p* < 0.05, ** *p* < 0.01, *** *p* < 0.001 vs. normoxia value. # *p* < 0.05, ## *p* < 0.01, ### *p* < 0.001 vs. pre-memantine state (n = 6 for APP+ and APP−).

Rivastigmine administration before hypercapnic stimulus both in APP+ and APP− mice evoked a decrease in all ventilatory parameters compared with the untreated state (Table 5, Figure 5A,B). The minute ventilation response to CO_2_ was reduced by 70.5% in APP+ mice and by 78.8% in APP− mice. Hypercapnic response reactivity after premedication with rivastigmine was significantly reduced for minute ventilation (Figure 5) and frequency of breathing (Table 5) in both groups of mice.

### 2.4. Acetylcholinesterase Activity in Different Brain Structures

**Figure 6:**
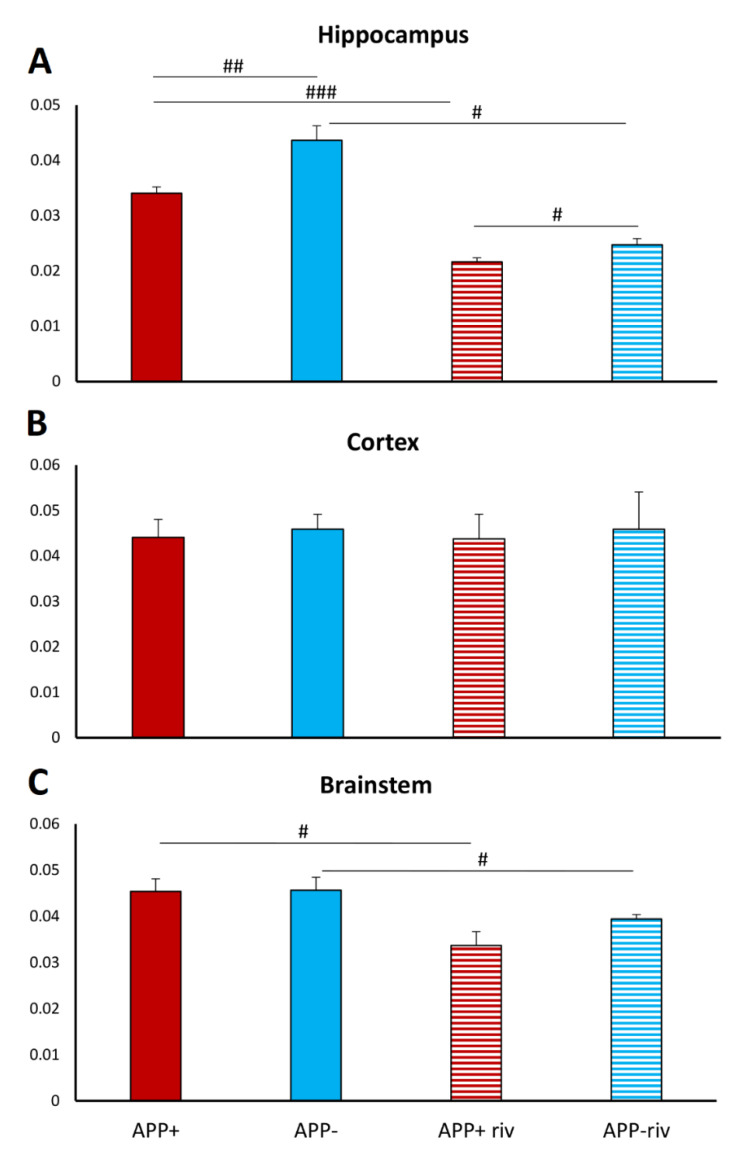
Acetylcholinesterase activity in hippocampus (**A**), cortex (**B**), and brainstem (**C**) in APP+ and APP− mice untreated (solid bars) and treated (striated bars) with rivastigmine. Data are expressed in mU min^−1^ mg^−1^ and presented as mean ± SEM. # *p* < 0.05, ## *p*< 0.01, ### *p*< 0.01 (n = 6 per each bar).

Acetylcholinesterase activity in the hippocampus, cortex, and brainstem was evaluated in both groups of rivastigmine-treated and untreated mice (1.5 mg/kg). The results showed changes in AChE activity in the hippocampus and brainstem (Figure 6A–C). APP+ mice had 22% less AChE activity in the hippocampus than healthy mice, and this activity was further reduced after rivastigmine administration in both groups (Figure 6A). After administration of rivastigmine, a 13% difference in enzyme activity was observed between APP− and APP+ mice, which was similar to the untreated condition (Figure 6A). Of interest are the results obtained in the brainstem analysis, where a reduction in AChE activity was observed after rivastigmine treatment in APP+ (26%) and APP− (14%) mice (Figure 6C).

## 3. Discussion

Our study is the first in a transgenic model of Alzheimer’s disease to address respiratory responses to the stressful stimuli of hypoxia and hypercapnia. The main finding of our study was a notable increase in the ventilatory response to hypercapnia and no difference in the ventilatory response to hypoxia in APP+ mice, compared with their controls. In contrast, respiratory parameters during air breathing were comparable in both groups.

A previous study in a streptozotocin (STZ)-induced model of AD displayed blunted ventilatory response to hypoxia with concomitant astrogliosis in the commissural part of the nucleus tractus solitarii (NTS) [12]. The authors speculated that astrogliosis of the primary site for afferent input from hypoxia-sensing carotid bodies (CB) [17] could contribute to inflammation and reactive oxygen species (ROS) formation and to impaired NTS function [12]. Two years later, they confirmed a decrease in the ventilatory response to hypoxia and showed a significant decrease in c-Fos labeling as a marker of neuronal activation in the caudal/medial NTS, rostral ventral respiratory group, and Bötzinger complex [13]. They concluded that the lower c-Fos staining may be due to local neuronal dysregulation of energy metabolism, which was demonstrated in the STZ model in the form of brain glucose levels dysregulation and desensitization of neuronal insulin receptors [18].

A study by another group, also on the STZ model, showing an increase in response to hypercapnia and no difference in response to hypoxia [14], is similar to our results. The enhanced CO_2_ sensitivity was attributed to significantly increased Aβ expression in the locus coeruleus (LC), an important chemosensitive area in the brainstem. Although the key pathological features of AD in the STZ model, such as neuroinflammation, oxidative stress, accumulation of phosphorylated tau and Aβ peptide, and synaptic damage in animal brains, leading to behavioral changes, point to its importance in translational research [19,20], when we consider the respiratory studies available to date, the differences between the studies presented are not consensual. Perhaps differences between experimental conditions such as different STZ doses (2 vs. 3 mg/kg), different rat strains (Wistar vs. Sprague Dawley) or time intervals after STZ injection (2 to 4 weeks), as well as different periods of exposure to hypoxia or hypercapnia may contribute to the differences between studies. Nevertheless, the question arises whether the streptozotocin model is appropriate for studying respiratory impairment in AD.

Since there are no more in vivo experimental studies on this problem apart from the literature cited above, we decided to investigate the respiratory responses to hypoxia and hypercapnia for the first time in a transgenic mouse model with a mutation of the amyloid β precursor protein (APP), which is also carried by individuals with familial Alzheimer’s disease. Mice exhibit a progressive appearance of senile plaques enriched in the neurotoxic Aβ42 isoform, dystrophic neurites, synaptic loss, and astrogliosis [21,22]. Hence, the enhanced response to hypercapnia present in our transgenic mice could possibly be related to the presence of Aβ in the brain regions responsible for CO_2_ sensing, as indicated in the study by Vincente et al. [14]. LC, one of the most relevant chemoreceptive areas, is damaged very early in AD and shows increased noradrenaline (NA) metabolism [23,24]. Therefore, we cannot exclude that the remaining LC neurons unaffected by Aβ may compensate for the reduced NA and enhance the respiratory response to hypercapnia.

However, we did not study neurodegeneration in LC or the level of Aβ in it and focused on disruptions in the glutamatergic and cholinergic systems. Therefore, we tested whether drugs used as standard treatment for cognitive impairment in Alzheimer’s disease, affecting both systems, have an effect on breathing, an issue that has not been studied before. In a mouse transgenic model of AD with overexpression of various APP mutations in the brain, disruption of glutamatergic signaling affecting learning and memory has been postulated [15]. Aβ-induced oxidative stress causes dysregulation of the glutamatergic neurotransmission system, i.e., accumulation of extracellular glutamate and increased activation of NMDA receptors, resulting in excitotoxicity [25,26]. The main molecule used in NMDA-based therapy to treat moderate-to-severe forms of AD is memantine. To see if this drug used to treat cognitive function affects breathing, we investigated the effect of memantine administration on air breathing and responses to hypoxia and hypercapnia in APP+ mutants and their APP− controls. We found that memantine at the dose used in our study had no significant effect on the respiration of both groups of mice, including responses to hypoxia and hypercapnia. This may seem surprising since glutamatergic transmission and NMDA receptors have been repeatedly shown to mediate critical components of respiratory control [27,28,29,30]. For instance, glutamatergic excitations in pre-Bötzinger (preBötC) and Bötzinger (BötC) complexes have been shown to be essential for the regular respiratory rhythm and respiratory phase timing, respectively, as NMDA receptor antagonism resulted in a decrease in inspiratory and expiratory duration, as well as peak phrenic amplitude and ultimately apnea [30]. Furthermore, simultaneous microinjection of NMDA and non-NMDA receptor antagonists into the commissural nucleus of the NTS were able to abolish the ventilatory responses to carotid body stimulation [27].

The role of NMDA receptors in the respiratory response to peripheral and central chemoreceptor stimulation was previously investigated in conscious rats without AD, in which, after blockading NMDA receptors with MK-801, a slightly reduced ventilation during eucapnia, an attenuated response to hypoxia, and unaltered respiratory response to hypercapnia were observed [31]. A similar result is an increase in respiratory rates in normoxia, which did not reach significance in our study. MK-801, however, is an antagonist with higher binding affinity, given intravenously at twice the dose, which certainly could have made a difference. We stayed with the memantine dose of 2.5 mg because higher doses cause behavioral changes that make it impossible to obtain reliable data on respiratory parameters in awake animals. We can conclude that, in our study, intraperitoneal administration of memantine, more resembling the longer absorption with oral administration in humans, had no significant effect on air breathing and ventilatory responses to chemoreceptor stimulation in both AD model and control mice.

Other pathological changes in mice with Aβ overexpression include amyloid plaques near acetylcholinesterase-immunoreactive structures, deafferentation of the cholinergic system, and shrinkage of cholinergic cells [32]. Addressing changes in the cholinergic system, we examined the effects of rivastigmine administration on ventilatory responses to hypoxia and hypercapnia. Rivastigmine, which acts by increasing the concentration of acetylcholine (ACh) in the synaptic cleft [33], depressed substantially resting ventilation and was very effective in reducing chemoreflex respiratory response to hypercapnia mostly due to a decrease in the frequency of breathing, irrespective of the mice genotype. In the case of hypoxia, a reduction in augmented minute ventilation was observed only in APP+ mice. We also found that the cholinesterase inhibitor (AChE), at a dose used to treat dementia in AD, was potent in reducing the increased hypercapnic ventilatory response well below the level present in control APP^_^ mice. We also revealed that APP+ mice exhibited lower AChE activity but only in the hippocampus, compared with healthy mice, and treatment with rivastigmine reduced the enzyme activity in both groups to a similar extent in the hippocampus and brainstem. The lack of enzyme inhibition after rivastigmine in the cortex was probably due to large standard deviations and differences between animals. In contrast to the brainstem and hippocampus, where whole structures were homogenized, in the case of the cortex, only a fragment of it was analyzed. Efforts were made to take it from the frontal cortex area, although this may not always have been an identical fragment.

The cholinergic system is involved in respiratory control, including central chemosensitivity, state-dependent modulation of breathing, and respiratory motor output [34,35,36,37]. The retrotrapezoid nucleus (RTN) contributing to chemical respiratory drive (CO_2_/H^+^) [38] receives cholinergic input from the post-inspiratory complex (PiCO) and pedunculopontine tegmental nucleus (PPTg), and stimulation of PPTg has been shown to increase respiratory activity, in part, by cholinergic activation of chemosensitive RTN [36]. In addition, cholinergic innervation is also present in the parafacial respiratory group (pFRG) where muscarinic transmission contributes to neuronal excitation and promotes recruitment of abdominal muscles and active expiratory flow [35]. Numerous cholinergic neurons are localized in the medullary reticular formation close to the ventral medullary area [39]. Hypoglossal nerve motoneurons [40], pre-Bötzinger complex (respiratory rhythm generator), and phrenic motoneurons receive cholinergic input [39].

Most physiologic studies indicate an excitatory effect of cholinergic system stimulation on respiratory function. For instance, acetylcholine applied in the commissural subnucleus of the NTS or intrathecally injected at the spinal C4 segment promotes an increase in the phrenic nerve activity [41,42]. Activation of nicotinic ACh receptors in the preBötC plays a crucial role in respiratory rhythm generation, resulting in an increase in respiratory frequency [39]. Administration of cholinergic agents to the ventral–lateral surface of the medulla, rich in central chemoreceptors, increases phrenic and hypoglossal activity under hypercapnic breathing [43]. The inhibition of ACh breakdown and increase in its concentration in the brain, obtained in our study, resulting in respiratory depression, does not quite match the study cited above. Nevertheless, it was previously shown that injection of ACh into the ventricles of the brain in anesthetized cats induced transient respiratory depression [44]. Additionally, in the study by Sahin et al. [45], in addition to a modest increase in respiratory rate, central and peripheral administration of ACh in dogs caused an initial decrease in tidal volume, leading to a significant decrease in minute ventilation of approximately 40%, similar to our results.

Acetylcholine injected into the intermediate portion of the NTS decreased phrenic nerve activity, whereas increased activity was observed when ACh was administered into the commissural NTS [41]. It, therefore, appears that the effect of ACh may depend on the site of administration. The depressant effect observed in our study was the result of a nonselective increase in this transmitter at the synaptic cleft throughout the whole brain.

## 4. Materials and Methods

### 4.1. Animals

All experimental procedures were approved by the local Ethical Committee for Animal Experimentation (Warsaw, Poland) and conducted in accordance with the European Union Directive 63/2010 and the respective Polish law regulations on the use and care of laboratory animals. In total, 24 adult female mice aged 12 months were used; 12 FVB-Tg (Thy1; APP LD2/B6) overexpressing human AβPP with V717I (“London”) mutation under the control of a fragment of thy 1 promoter (APP+), and 12 mice that do not inherit the transgene were treated as controls (APP−). The mice were maintained and held in the animal house of the Mossakowski Medical Research Institute (Warsaw, Poland) under specific pathogen-free conditions, controlled temperature, and humidity, with 12 h light–12 h dark cycle, and water and food available ad libitum.

### 4.2. Plethysmography Measurements

Ventilation and respiratory parameters were recorded in conscious unrestrained mice using a whole-body rodent plethysmography chamber (Buxco Electronics Inc., Wilmington, NC, USA), as described previously [46]. The pressure signal was amplified, filtered, recorded, and analyzed with data analysis software (Biosystem XA for Windows, SFT3410 230 v2.9; Buxco Electronics, Wilmington, NC, USA) generating tidal volume (V_T_, mL) and breathing frequency (F, breaths min^−1^). Tidal volume was calculated using the approach of Epstein et al. [47]. Minute ventilation (V_E_, mL min^−1^, BTPS) was determined as a product of tidal volume and breathing frequency. V_T_ and V_E_ were normalized to body weight (mL g^−1^ and mL g^−1^ min^−1^, respectively). Rectal temperature was measured before and at the end of the experiments. All experiments were performed at room temperature (22–24 °C). Each mouse was placed in the chamber and left for 30 min of acclimatization before measurements of baseline were performed. The chamber was ventilated continuously with atmospheric air at a rate of 500 mL min^−1^ to prevent CO_2_ accumulation. Volume calibration was performed before each experiment by injecting a known volume of air inside the chamber.

Acute hypoxia was induced by rapid flushing of a gas mixture containing 8% of O_2_ in N_2_. Acute hypercapnia was achieved by flushing with a mixture of 7% CO_2_ (79% N_2_ and 21% O_2_). Ventilation and its response to inspired hypoxia or hypercapnia were registered before and after drug administration. After 30 min of adaptation to breathing with the chamber air. Pulmonary ventilation was taken as the baseline level of ventilation and recorded for 1 min before the introduction of hypoxia or hypercapnia. Ventilation during 3 min of hypoxic or hypercapnic stimulus and 5 min after switching to the air breathing was recorded. A period of 30 s of breathing preceding hypoxia was calculated as a control normoxic breathing. The three-minute breathing period in hypoxia or hypercapnia was averaged.

### 4.3. Drugs and Treatment Schedule: Treatment with Rivastigmine and Memantine before and after Hypoxia and Hypercapnia in Unrestrained Mice

The compounds used in this study were rivastigmine tartrate (Sigma, Poznań, Poland) a reversible acetylcholinesterase inhibitor indicated for the treatment of mild-to-moderate dementia associated with Alzheimer’s disease and Parkinson’s disease, and memantine hydrochloride (Sigma, Poznań, Poland), a noncompetitive NMDA receptor antagonist used for the treatment of moderate-to-severe Alzheimer-type dementia. The dose of rivastigmine 1.5 mg/kg was identical to that used in patients to treat cognitive impairment [48]; in the case of memantine, a low dose of 2.5 mg/kg was selected based on our preliminary experiments, showing that higher doses affected behavior, as well as based on previous animal research [49]. Both drugs were prepared from powder freshly before each injection and dissolved in 0.9% NaCl saline solution.

**Figure 7:**
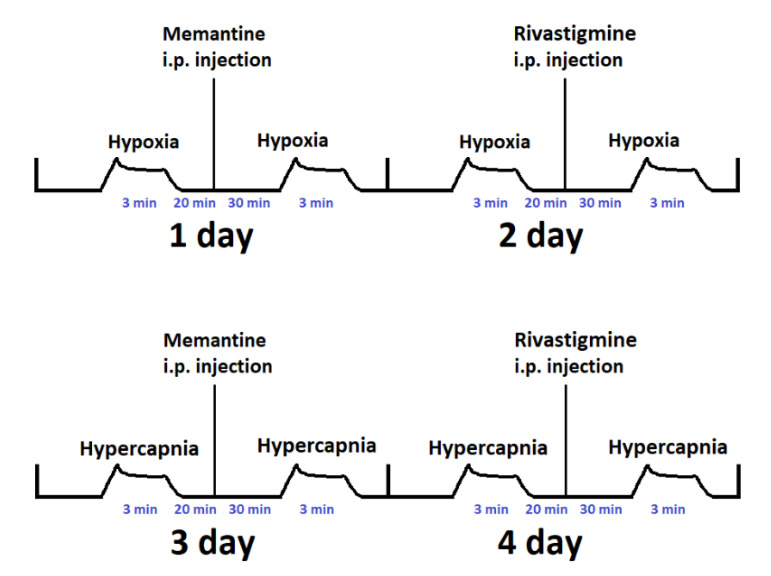
A detailed description of the stimulus exposures (hypoxia or hypercapnia) and compound administration performed (memantine or rivastigmine).

The experimental scheme, presented in Figure 7, was as follows:

In the first group of six APP+ and six APP− mice, respiratory responses to hypoxia (first day) and hypercapnia (next day) were examined without pharmacological interventions. After the experiments were completed and the animals were euthanized, their brains were collected for further analysis.

In the second group of six APP+ and six APP− mice, on the first day of the experiment, 20 min after control hypoxia, animals received intraperitoneal memantine at a dose of 2.5 mg/kg, and 30 min after injection, acute hypoxia was reapplied. On the following second day, rivastigmine at a dose of 1.5 mg/kg was administered 30 min before the second hypoxia exposure, with a similar regimen. On the third and fourth days, compound administration was repeated as on the first and second days, except that this time the stimulus preceding and following memantine or rivastigmine injection was hypercapnia. To limit the number of animals used in the study, we administered compounds to the same animals but only once on a given day. The intervals between successive administrations were at least 24 h. The half-life of rivastigmine is short at 3–4 h, while the half-life of memantine is about 60 h, so we injected memantine a second time after two days [50,51]. Moreover, the results after the application of the compounds were always related to the control respiration, measured on a given day, which did not differ significantly from one day to the next, indicating that the compounds had no long-term effect on respiration. After the experiments were completed and the animals euthanized, their brains were collected for further analysis.

### 4.4. The Acetylcholinesterase (AChE) Activity Assay *(Ellman’s Assay)*

After the experiments were completed, mouse brains were isolated and sections of the prefrontal cortex, hippocampus, and brainstem were collected. Frozen brain tissues were homogenized in lysis buffer (50mM TRIS-HCl solution with 150 mM NaCl, 1% Triton X-100, pH = 8) using an ultrasound homogenizer. Additionally, 20 mg tissue/mL of buffer was used. The prepared homogenates were centrifuged at 14000 rpm for 10 min at 4 °C. Then, the supernatant was used for AChE activity determination. The AChE activity in examined samples was measured quantitatively via Ellman’s method [52], with modifications, using a 96-well microplate reader (Epoch. BioTek, Winooski, VT, USA). The mixture was prepared by mixing 0.4 mL supernatant with 2.6 mL of DTNB solution (10 mM 5.5 dithiobis 2-nitrobenzoic acid in phosphate buffer). Next, 155 µL of the mixture was transferred to a 96-well plate in triplicate for each sample. After that, the plate was incubated for 10 min at 37 °C. After incubation, 10 µL of ACTI (7.5 mM acetylthiocholine iodide in distilled water) was added to each well, and absorbance was measured at 412 nm for 10 min at 1 min intervals. AChE activity was determined using a calibration curve performed at each measurement using acetylcholinesterase from *Electrophorus electricus* (electric eel, Sigma, Poznań, Poland) and expressed in mU min^−1^ mg^−1^.

### 4.5. Statistics

Data are reported as means ± SEM. Statistical analysis was performed using Statistica version 12 (StatSoft, Kraków, Poland). Normality of distributions was tested with Shapiro–Wilk test. Statistical analysis was performed using nonparametric statistics Kruskal–Wallis ANOVA, followed by the Mann–Whitney U test for comparison of the respiratory parameters between groups. For comparison within the group, Wilcoxon’s signed-rank test or Student’s *t*-test was used. For each analysis, *p* < 0.05 was taken to indicate a significant difference.

## 5. Conclusions

Transgenic mice with overproduction of amyloid-β showed augmented ventilatory response to hypercapnia, compared with control littermates. This may indicate that, in this genetic model of early-onset AD, there is an impairment in the chemoreceptive respiratory nuclei, resulting in increased sensitivity to CO_2_. The acetylcholinesterase inhibitor rivastigmine is a potent agent that reduces normoxic ventilation and hypercapnic ventilatory response in all mice. Therefore, we suggest that this drug should be used with caution in patients with respiratory problems.

## Figures and Tables

**Table 1:** Tidal volume (V_T_) and frequency of breathing (F) baseline values during air breathing (normoxia), hypoxia, and hypercapnia in APP+ and APP− mice. In the two right columns, V_T_ and F during the respiratory response to hypoxia and hypercapnia are expressed as a percentage of baseline values in normoxia.

HYPOXIA				
	Baseline Data		Percentage of Baseline	
Group	Normoxia	Hypoxia	Normoxia	Hypoxia
Tidal volume (mL g^−1^)				
APP−	13.26 (±0.09)	17.86 (±0.57) *	100 (±0)	136.26 (±6.37) **
APP+	15.09 (±0.66)	20.081 (±1.57) **	100 (±0)	129.1015 (±14.56)
Frequency of breathing (breath min^−1^)				
APP−	150.61 (±12.71)	278.50 (±11.11) ***	100 (±0)	188.53 (±11.80) ***
APP+	152.89 (±6.46)	313.35 (±8.7) *** #	100 (±0)	206.31 (±10.73) ***
HYPERCAPNIA				
	Baseline data		Percentage of baseline	
Group	Normoxia	Hypercapnia	Normoxia	Hypercapnia
Tidal volume (mL g^−1^)				
APP−	13.99 (±0.97)	21.85 (±2.25) **	100 (±0)	155.24 (±10.08) **
APP+	15.30 (±0.48)	22.12 (±3.64)	100 (±0)	143.77 (±21.72)
Frequency of breathing (breath min^−1^)				
APP−	147.17 (±10.71)	203.08 (±18.01) ***	100 (±0)	137.5 (±4.11) ***
APP+	142.41 (±8.99)	253.83 (±16.84) *** #	100 (± 0)	179.64 (±11.84) *** ##

The data are presented as mean ± SEM; * *p* < 0.05. ** *p* < 0.01. *** *p* < 0.001 vs. baseline normoxia value. # *p* < 0.05, ## *p* < 0.01 vs. APP− value (n = 6 for APP+ and APP−).

**Table 2:** Tidal volume (V_T_) and frequency of breathing (F) baseline values during air breathing (normoxia) and hypoxia in APP+ and APP− mice before and after memantine injection. In the two right columns, V_T_ and F during the respiratory response to hypoxia are expressed as a percentage of baseline values in normoxia.

	Baseline Data		Percentage of Baseline	
Group	Normoxia	Hypoxia	Normoxia	Hypoxia
Tidal volume (mL g^−1^)				
APP−	11.14 (±0.56)	13.46 (±0.85) **	100 (±0)	120.7 (±3.87) **
APP− memantine	10.97 (±0.79)	14.94 (±0.79) ***	100 (±0)	137.9 (±7.60) **
APP+	12.9 (±0.59) #	17.09 (±1.12) * #	100 (±0)	133.6 (±10.07) *
APP+ memantine	12.72 (±0.77)	18.96 (±0.94) ** ##	100 (±0)	151.1 (±10.69) **
Frequency of breathing (breath min^−1^)				
APP−	165.11 (±5.44)	316.7 (±13.03) ***	100 (±0)	192.33 (±8.3) ***
APP− memantine	208.97 (±21.82)	328.68 (±6.74) *	100 (±0)	162.78 (±13.37) **
APP+	157.98 (±5.13)	319.88 (±12.09) ***	100 (±0)	204.24 (±13.4) ***
APP+ memantine	168.94 (±9.80)	328.6 (±15.41) ***	100 (±0)	199.44 (±21.36) ***

The data are presented as mean ± SEM; * *p* < 0.05, ** *p* < 0.01, *** *p* < 0.001 vs. baseline normoxia value. # *p* < 0.05, ## *p* < 0.01, vs. APP− value. (n = 6 for APP+ and APP−).

**Table 3:** Tidal volume (V_T_) and frequency of breathing (F) baseline values during air breathing (normoxia) and hypercapnia in APP+ and APP− mice before and after memantine injection. In the two right columns, V_T_ and F during the respiratory response to hypercapnia are expressed as a percentage of baseline values in normoxia.

	Baseline Data		Percentage of Baseline	
Group	Normoxia	Hypercapnia	Normoxia	Hypercapnia
Tidal volume (mL g^−1^)				
APP−	10.36 (±0.66) -	16.89 (±1.98) **	100 (±0)	161.76 (±14.39) **
APP− memantine	11.09 (±0.43)	17.66 (±1.81) **	100 (±0)	157.63 (±11.33) **
APP+	12.43 (±0.28) #	18.64 (±1.77) *	100 (±0)	158.57 (±5.88) ***
APP+ memantine	13.78 (±1.36) #	21.01 (±1.57) *	100 (±0)	155.37 (±13.07) **
Frequency of breathing (breath min^−1^)				
APP−	159.09(±8.15) -	319.26 (±23.42) ***	100 (±0)	200.55 (±10.47) *
APP− memantine	184.73 (±13.1)	372.08 (±21.35) ***	100 (±0)	205.04 (±17.58) **
APP+	141.37 (±5.37) +	384.19 (±22.87) ***	100 (±0)	265.43 (±14.04) *** ##
APP+ memantine	155.03 (±0.01)	441.21 (±18.63) *** #	100 (±0)	285.44 (±6.36) *** ###

The data are presented as mean ± SEM; * *p* < 0.05, ** *p* < 0.01, *** *p* < 0.001 vs. baseline normoxia value. # *p* < 0.05, ## *p* < 0.01, ### *p* < 0.001 vs. respective APP− value. - *p* < 0.05 vs. APP− memantine. + *p* < 0.05 vs. APP+ memantine (n = 6 for APP+ and APP−).

**Table 4:** Tidal volume (V_T_) and frequency of breathing (F) baseline values during air breathing (normoxia) and hypoxia in APP+ and APP− mice before and after rivastigmine injection. In the two right columns, V_T_ and F during the respiratory response to hypoxia are expressed as a percentage of baseline values in normoxia.

	Baseline Data		Percentage of Baseline	
Group	Normoxia	Hypoxia	Normoxia	Hypoxia
Tidal volume (mL g^−1^)				
APP−	10.51 (±0.75) ---	12.82 (±0.91) *** --	100 (±0)	122.15 (±3.21) *** -
APP− rivastigmine	5.54 (±0.31)	8.05 (±0.57) **	100 (±0)	146.10 (±9.19) **
APP+	13.24 (±0.62) # +++	18.05 (±0.81) *** ### +++	100 (±0)	136.62 (±4.13) *** #
APP+ rivastigmine	9.16 (±0.53) ###	12.37 (±0.24) ** ##	100 (±0)	137.07 (±8.62) **
Frequency of breathing (breath min^−1^)				
APP−	167.5 (±3.71) -	323.86 (±7.16) *** ---	100 (±0)	193.73 (±5.99) *** --
APP− rivastigmine	141.12 (±10.04)	214.26 (±6.15) ***	100 (±0)	154.14 (±8.78) *
APP+	151.38 (±3.63) + ##	306.56 (±9.53) *** +++	100 (±0)	203.11 (±8.64) *** +
APP+ rivastigmine	128.08 (±7.96)	210.57 (±6.92) ***	100 (±0)	165.97 (±6.43) ** #

The data are presented as mean ± SEM; * *p* < 0.05, ** *p* < 0.01, *** *p* < 0.001 vs. baseline normoxia value, # *p* < 0.05, ## *p* < 0.01, ### *p* < 0.001 vs. APP− value, + *p* < 0.05, +++ *p* < 0.001 vs. APP+ rivastigmine, - *p* < 0.05, -- *p* < 0.01, --- *p* < 0.001 vs. APP− rivastigmine (n = 6 for APP+ and APP).

**Table 5:** Tidal volume (V_T_) and frequency of breathing (F) baseline values during air breathing (normoxia) and hypercapnia in APP+ and APP− mice before and after rivastigmine injection. In the two right columns, V_T_ and F during the respiratory response to hypercapnia are expressed as a percentage of baseline values in normoxia.

	Baseline Data		Percentage of Baseline	
Group	Normoxia	Hypercapnia	Normoxia	Hypercapnia
Tidal volume (mL g^−1^)				
APP−	11.09 (±1.44) --	16.16 (±2.19) ** --	100 (±0)	146.08 (±10.67) ** --
APP− rivastigmine	6.169 (±0.72)	7.245 (±0.98) *	100 (±0)	116.64 (±3.38) **
APP+	12.25 (±0.75) +	19.3 (±01.59) ** ++	100 (±0)	160.55 (±17.84) *
APP+ rivastigmine	9.12 (±0.83) #	11.038 (±0.84) ##	100 (±0)	123.18 (±10.14)
Frequency of breathing (breath min^−1^)				
APP−	162.22 (±5.28) -	340.64 (±24.83) *** -	100 (±0)	211.4 (±18.23) ** --
APP− rivastigmine	131.97 (±6.89)	156.96 (±16.36) *	100 (±0)	118.78 (±9.71)
APP+	143.23 (±6.00)	373.83 (±19.19) * +	100 (±0)	260.7 (±5.57) *** # +++
APP+ rivastigmine	148.43 (±12.47)	194.96 (±14.01) * #	100 (±0)	135.63 (±14.56) *

The data are presented as mean ± SEM; * *p* < 0.05, ** *p* < 0.01. *** *p* < 0.001 vs. baseline normoxia value, # *p* < 0.05. ## *p* < 0.01 vs. APP− value, + *p* < 0.05, ++ *p* < 0.01, +++ *p* < 0.001 vs. APP+ rivastigmine, - *p* < 0.05, -- *p* < 0.01 vs. APP− rivastigmine, (n = 6 for APP+ and APP−).

## Data Availability

The data used to support the findings of this study are available from the corresponding author upon request.

## References

[1] Burns A., Iliffe S. (2009). Alzheimer’s disease. BMJ.

[2] Wilson R.S., Segawa E., Boyle P.A., Anagnos S.E., Hizel L.P., Bennett D.A. (2012). The natural history of cognitive decline in Alzheimer’s disease. Psychol. Aging.

[3] Yeh S.J., Chung M.H., Chen B.S. (2021). Investigating Pathogenetic Mechanisms of Alzheimer’s Disease by Systems Biology Approaches for Drug Discovery. Int. J. Mol. Sci..

[4] Rajmohan R., Reddy P.H. (2017). Amyloid-Beta and Phosphorylated Tau Accumulations Cause Abnormalities at Synapses of Alzheimer’s disease Neurons. J. Alzheimers Dis..

[5] Fagiani F., Lanni C., Racchi M., Pascale A., Govoni S. (2019). Amyloid-β and Synaptic Vesicle Dynamics: A Cacophonic Orchestra. J. Alzheimers Dis..

[6] Bekdash R.A. (2021). The Cholinergic System, the Adrenergic System and the Neuropathology of Alzheimer’s Disease. Int. J. Mol. Sci..

[7] Cuadrado-Tejedor M., Pérez-González M., Alfaro-Ruiz R., Badesso S., Sucunza D., Espelosin M., Ursúa S., Lachen-Montes M., Fernández-Irigoyen J., Santamaria E. (2021). Amyloid-Driven Tau Accumulation on Mitochondria Potentially Leads to Cognitive Deterioration in Alzheimer’s Disease. Int. J. Mol. Sci..

[8] Reynolds C.F., Kupfer D.J., Taska L.S., Hoch C.C., Sewitch D.E., Restifo K., Spiker D.G., Zimmer B., Marin R.S., Nelson J. (1985). Sleep apnea in Alzheimer’s dementia: Correlation with mental deterioration. J. Clin. Psychiatry.

[9] Brunnström H.R., Englund E.M. (2009). Cause of death in patients with dementia disorders. Eur. J. Neurol..

[10] Sanches V.S., Santos F.M., Fernandes J.M., Santos M.L., Müller P.T., Christofoletti G. (2014). Neurodegenerative disorders increase decline in respiratory muscle strength in older adults. Respir. Care..

[11] Emamian F., Khazaie H., Tahmasian M., Leschziner G.D., Morrell M.J., Hsiung G.Y., Rosenzweig I., Sepehry A.A. (2016). The Association Between Obstructive Sleep Apnea and Alzheimer’s Disease: A Meta-Analysis Perspective. Front. Aging Neurosci..

[12] Ebel D.L., Torkilsen C.G., Ostrowski T.D. (2017). Blunted Respiratory Responses in the Streptozotocin-Induced Alzheimer’s Disease Rat Model. J. Alzheimers Dis..

[13] Brown A.G., Thapa M., Hooker J.W., Ostrowski T.D. (2019). Impaired chemoreflex correlates with decreased c-Fos in respiratory brainstem centers of the streptozotocin-induced Alzheimer’s disease rat model. Exp. Neurol..

[14] Vicente M.C., Almeida M.C., Bícego K.C., Carrettiero D.C., Gargaglioni L.H. (2018). Hypercapnic and Hypoxic Respiratory Response During Wakefulness and Sleep in a Streptozotocin Model of Alzheimer’s Disease in Rats. J. Alzheimers Dis..

[15] Moechars D., Dewachter I., Lorent K., Reversé D., Baekelandt V., Naidu A., Tesseur I., Spittaels K., Haute C.V., Checler F. (1999). Early phenotypic changes in transgenic mice that overexpress different mutants of amyloid precursor protein in brain. J. Biol. Chem..

[16] Van Dorpe J., Smeijers L., Dewachter I., Nuyens D., Spittaels K., Van Den Haute C., Mercken M., Moechars D., Laenen I., Kuiperi C. (2000). Prominent cerebral amyloid angiopathy in transgenic mice overexpressing the london mutant of human APP in neurons. Am. J. Pathol..

[17] Barnett W.H., Abdala A.P., Paton J.F., Rybak I.A., Zoccal D.B., Molkov Y.I. (2017). Chemoreception and neuroplasticity in respiratory circuits. Exp. Neurol..

[18] Grieb P. (2016). Intracerebroventricular Streptozotocin Injections as a Model of Alzheimer’s Disease: In Search of a Relevant Mechanism. Mol. Neurobiol..

[19] Kamat P.K. (2015). Streptozotocin induced Alzheimer’s disease like changes and the underlying neural degeneration and regeneration mechanism. Neural. Regen. Res..

[20] Ravelli K.G., Rosário B.D., Camarini R., Hernandes M.S., Britto L.R. (2017). Intracerebroventricular Streptozotocin as a Model of Alzheimer’s Disease: Neurochemical and Behavioral Characterization in Mice. Neurotox. Res..

[21] Masliah E., Sisk A., Mallory M., Mucke L., Schenk D., Games D. (1996). Comparison of neurodegenerative pathology in transgenic mice overexpressing V717F beta-amyloid precursor protein and Alzheimer’s disease. J. Neurosci..

[22] Roher A.E., Kokjohn T.A., Esh C., Weiss N., Childress J., Kalback W., Luehrs D.C., Lopez J., Brune D., Kuo Y.M. (2004). The human amyloid-beta precursor protein770 mutation V717F generates peptides longer than amyloid-beta-(40-42) and flocculent amyloid aggregates. J. Biol. Chem..

[23] Heneka M.T., Nadrigny F., Regen T., Martinez-Hernandez A., Dumitrescu-Ozimek L., Terwel D., Jardanhazi-Kurutz D., Walter J., Kirchhoff F., Hanisch U.K. (2010). Locus ceruleus controls Alzheimer’s disease pathology by modulating microglial functions through norepinephrine. Proc. Natl. Acad. Sci. USA.

[24] Feinstein D.L., Kalinin S., Braun D. (2016). Causes, consequences, and cures for neuroinflammation mediated via the locus coeruleus: Noradrenergic signaling system. J. Neurochem..

[25] Kandimalla R., Reddy P.H. (2017). Therapeutics of Neurotransmitters in Alzheimer’s Disease. J. Alzheimers Dis..

[26] Bukke V.N., Archana M., Villani R., Romano A.D., Wawrzyniak A., Balawender K., Orkisz S., Beggiato S., Serviddio G., Cassano T. (2020). The Dual Role of Glutamatergic Neurotransmission in Alzheimer’s Disease: From Pathophysiology to Pharmacotherapy. Int J. Mol. Sci..

[27] Vardhan A., Kachroo A., Sapru H.N. (1993). Excitatory amino acid receptors in commissural nucleus of the NTS mediate carotid chemoreceptor responses. Am. J. Physiol..

[28] Kaczyńska K., Szereda-Przestaszewska M., Chrapusta S.J. (2006). Non-vagal apnea evoked by intra-common carotid artery injection of N-methyl-D-aspartic acid (NMDA) in anesthetized rats. Acta Neurobiol. Exp..

[29] Pamenter M.E., Nguyen J., Carr J.A., Powell F.L. (2014). The effect of combined glutamate receptor blockade in the NTS on the hypoxic ventilatory response in awake rats differs from the effect of individual glutamate receptor blockade. Physiol. Rep..

[30] Cook-Snyder D.R., Miller J.R., Navarrete-Opazo A.A., Callison J.J., Peterson R.C., Hopp F.A., Stuth E.A.E., Zuperku E.J., Stucke A.G. (2019). The contribution of endogenous glutamatergic input in the ventral respiratory column to respiratory rhythm. Respir. Physiol. Neurobiol..

[31] Ohtake P.J., Torres J.E., Gozal Y.M., Graff G.R., Gozal D. (1998). NMDA receptors mediate peripheral chemoreceptor afferent input in the conscious rat. J. Appl. Physiol..

[32] Bronfman F.C., Moechars D., Van Leuven F. (2000). Acetylcholinesterase-positive fiber deafferentation and cell shrinkage in the septohippocampal pathway of aged amyloid precursor protein london mutant transgenic mice. Neurobiol. Dis..

[33] Khoury R., Rajamanickam J., Grossberg G.T. (2018). An update on the safety of current therapies for Alzheimer’s disease: Focus on rivastigmine. Ther Adv. Drug Saf..

[34] Sobrinho C.R., Kuo F.S., Barna B.F., Moreira T.S., Mulkey D.K. (2016). Cholinergic control of ventral surface chemoreceptors involves Gq/inositol 1,4,5-trisphosphate-mediated inhibition of KCNQ channels. J. Physiol..

[35] Boutin R.C.T., Alsahafi Z., Pagliardini S. (2017). Cholinergic modulation of the parafacial respiratory group. J. Physiol..

[36] Lima J.D., Sobrinho C.R., Falquetto B., Santos L.K., Takakura A.C., Mulkey D.K., Moreira T.S. (2019). Cholinergic neurons in the pedunculopontine tegmental nucleus modulate breathing in rats by direct projections to the retrotrapezoid nucleus. J. Physiol..

[37] Furuya W.I., Bassi M., Menani J.V., Colombari E., Zoccal D.B., Colombari D.S.A. (2020). Modulation of hypercapnic respiratory response by cholinergic transmission in the commissural nucleus of the solitary tract. Pflugers Arch..

[38] Guyenet P.G., Bayliss D.A. (2015). Neural Control of Breathing and CO2 Homeostasis. Neuron.

[39] Shao X.M., Feldman J.L. (2009). Central cholinergic regulation of respiration: Nicotinic receptors. Acta Pharmacol. Sin..

[40] Rukhadze I., Kubin L. (2007). Mesopontine cholinergic projections to the hypoglossal motor nucleus. Neurosci. Lett..

[41] Furuya W.I., Bassi M., Menani J.V., Colombari E., Zoccal D.B., Colombari D.S. (2014). Differential modulation of sympathetic and respiratory activities by cholinergic mechanisms in the nucleus of the solitary tract in rats. Exp. Physiol..

[42] Zhang R.X., Hui N. (1991). Effect of intrathecal injection of acetylcholine on phrenic nerve firing activity in rabbits. Sheng Li Xue Bao.

[43] Haxhiu M.A., Mitra J., Van Lunteren E., Bruce E.N., Cherniack N.S. (1984). Hypoglossal and phrenic responses to cholinergic agents applied to ventral medullary surface. Am. J. Physiol..

[44] Dikshit B.B. (1934). Action of acetylcholine on the brain and its occurrence therein. J. Physiol..

[45] Sahin G., Oruc T., Simsek G., Guner I. (1998). The effect of central and peripheral administration of acetylocholine and epinephrine on respiration. In. J. Physiol. Pharmacol..

[46] Andrzejewski K., Kaczyńska K., Zaremba M. (2017). Serotonergic system in hypoxic ventilatory response in unilateral rat model of Parkinson’s disease. J. Biomed. Sci..

[47] Epstein R.A., Epstein M.A.F., Haddad G.G., Mellins R.B. (1980). Practical implementation of the barometric method for measurement of tidal volume. J. Appl. Physiol..

[48] Gottwald M.D., Rozanski R.I. (1999). Rivastigmine, a brain-region selective acetylcholinesterase inhibitor for treating Alzheimer’s disease: Review and current status. Expert. Opin. Investig. Drugs.

[49] Réus G.Z., Valvassori S.S., Machado R.A., Martins M.R., Gavioli E.C., Quevedo J. (2008). Acute treatment with low doses of memantine does not impair aversive, non-associative and recognition memory in rats. Naunyn Schmiedebergs Arch. Pharmacol..

[50] Noetzli M., Eap C.B. (2013). Pharmacodynamic, pharmacokinetic and pharmacogenetic aspects of drugs used in the treatment of Alzheimer’s disease. Clin. Pharmacokinet..

[51] Valis M., Herman D., Vanova N., Masopust J., Vysata O., Hort J., Pavelek Z., Klimova B., Kuca K., Misik J. (2019). The Concentration of Memantine in the Cerebrospinal Fluid of Alzheimer’s Disease Patients and Its Consequence to Oxidative Stress Biomarkers. Front. Pharmacol..

[52] Ellman G.L., Courtney K.D., Andres V., Featherstone R.M. (1961). A new and rapid colorimetric determination of acetylcholinesterase activity. Biochem. Pharmacol..

